# Supplementary material for: Impact of Enniatin and Deoxynivalenol Co-Occurrence on Plant, Microbial, Insect, Animal and Human Systems: Current Knowledge and Future Perspectives
Source: Toxins (Basel). 2023 Apr 6;15(4):271. doi: 10.3390/toxins15040271 (PMC10144843; doi:10.3390/toxins15040271)
Supplement: Supplementary file 1 [file toxins-15-00271-s001.zip › toxins-2303383-supplementary.pdf]

Impact of enniatin and deoxynivalenol co-occurrence on plant, microbial, insect, animal and human systems: current knowledge and future perspectives” by **Irene Valenti [1], Francesco Tini [2,*], Milos Sevarika [2], Alessandro Agazzi [3], Giovanni Beccari [2], Ilaria Bellezza [4], Luisa Ederli [2], Silvia Grottelli [4], Matias Pasquali [1], Roberto Romani [2], Marco Saracchi [2], and Lorenzo Covarelli [2]**

[1] Department of Food, Environmental and Nutritional Sciences, University of Milan, 20133 Milan, Italy;
[2] Department of Agricultural, Food and Environmental Sciences, University of Perugia, 06121 Perugia, Italy;
[3] Department of Veterinary Medicine and Animal Sciences, University of Milan, 26900 Lodi, Italy;
[4] Department of Medicine and Surgery, University of Perugia, 06132 Perugia, Italy.

*Correspondence: francesco.tini@unipg.it

Supplementary Table S1: In vitro effects of ENNs on microorganisms.

* = not sensitivity at the highest test concentration; [a] = MIC value; [b] = IC_{50} value.

	Species/Cell line	Sensitivity	ENN type	Reference
Bacteria	*Cryptococcus neoformans* ATCC 90113	12.5 µg/ml [a]	ENA	195
		12.5 µg/ml [a]	ENA1	
		25 µg/ml [a]	ENB1	
	Clostridium perfringens CECT 4647	0.2 µg [a]	ENB	122
	Enterococcus faecium CECT 410	2000 µg [a]		
	Escherichia coli CECT 4782	>2000 µg[a]*		
	Listeria monocytogenes CECT 935	2000 µg [a]		
	Pseudomonas aeruginosa CECT 4628	2000 µg [a]		
	Salmonella enterica CECT 554	200 µg [a]		
	Shigella dysenteriae CECT 584	>2000 µg[a]*		
	Staphylococcus aureus CECT 240	>2000 µg[a]*		
	Staphylococcus aureus CECT 976	0.2 µg [a]		
	Yersinia enterocolitica CECT 4054	2000 µg [a]		
	Mycobacterium intracellulare ATCC 23068	50 µg/ml [a]	ENA	195
		50 µg/ml [a]	ENA1	
		>50 µg/ml[a]*	ENB1	
	Mycobacterium tubercolosis H37Ra	3.12 µg/ml [a]	ENB	120
		3.12 µg/ml [a]	ENB4	
		6.25 µg/ml [a]	ENC	
		6.25 µg/ml [a]	ENG	
		6.25 µg/ml [a]	ENH	
		6.25 µg/ml [a]	ENI	

	Mycobacterium tubercolosis H37Ra ATCC25177	2 μg/ml [a]	ENA1	119
	Mycobacterium tubercolosis H37Rv ATCC27294	1 μg/ml [a]		
	Mycobacterium bovis ATCC19210	2 μg/ml [a]		
	Mycobacterium bovis BGC ATCC35737	2 μg/ml [a]		
	Mycobacterium smegmatis mc² 155	8 μg/ml [a]		
	Escherichia coli ATCC25922	>100 μg/ml [a]		
	Klebsiella pneumonia CTCC46117	>100 μg/ml [a]		
	Listeria monocytogenes ATCC19115	>100 μg/ml [a]		
	Pseudomonas aeruginosa ATCC9027	>100 μg/ml [a]		
	Staphylococcus aureus ATCC75923	>100 μg/ml [a]		
	Bifidobacterium adolescentis CECT 5781	>1000 ng[a]*	ENJ1	127
	Bifidobacterium bifidum CECT 870T	10 ng [a]		
	Bifidobacterium breve CECT 4839T	100 ng [a]		
	Bifidobacterium longum CECT 4551	1000 ng [a]		
	Clostridium perfringens CECT 4647	100 ng [a]		
	Enterococcusfaecium CECT 410	10 ng [a]		
	Escherichia coli CECT 4782	>1000 ng[a]*		
	Lactobacillus animalis CECT 4060T	100 ng [a]		
	Lactobacillus casei CECT 475	1000 ng [a]		
	Lactobacillus casei subsp. casei CECT 475	1000 ng [a]		
	Lactobacillus casei rhamnosus CECT 278T	10 ng [a]		
	Lactobacillus plantarum CECT 220	10 ng [a]		
	Lactobacillus rhuminis CECT 4061T	1000 ng [a]		
	Listeria monocytogenes CECT 935	>1000 ng[a]*		
	Pseudomonas aeruginosa CECT 4628	>1000 ng[a]*		
	Salmonella enterica CECT 554	>1000 ng[a]*		
	Shigella dysenteriae CECT 584	100 ng [a]		
	Staphylococcus aureus CECT 976	1000 ng [a]		
	Staphylococcus aureus CECT 240	1000 ng [a]		
	Yersinia enterocolitica CECT 4054	100 ng [a]		
	Bifidobacterium adolescentis CECT 5781	1000 ng [a]	ENJ3	

	Bifidobacterium bifidum CECT 870T	10 ng [a]		
	Bifidobacterium breve CECT 4839T	>1000 ng[a*]		
	Bifidobacterium longum CECT 4551	10 ng [a]		
	Clostridium perfringens CECT 4647	1000 ng [a]		
	Enterococcusfaecium CECT 410	1000 ng [a]		
	Escherichia coli CECT 4782	1000 ng [a]		
	Lactobacillus animalis CECT 4060T	10 ng [a]		
	Lactobacillus casei CECT 475	100 ng [a]		
	Lactobacillus casei subsp. casei CECT 475	1000 ng [a]		
	Lactobacillus casei rhamnosus CECT 278T	1000 ng [a]		
	Lactobacillus plantarum CECT 220	1000 ng [a]		
	Lactobacillus rhuminis CECT 4061T	1000 ng [a]		
	Listeria monocytogenes CECT 935	>1000 ng[a*]		
	Pseudomonas aeruginosa CECT 4628	>1000 ng[a*]		
	Salmonella enterica CECT 554	>1000 ng[a*]		
	Shigella dysenteriae CECT 584	10 ng [a]		
	Staphylococcus aureus CECT 976	>1000 ng[a*]		
	Staphylococcus aureus CECT 240	10 ng [a]		
	Yersinia enterocolitica CECT 4054	>1000 ng[a*]		
	Bifidobacterium adolescentis 5871	>20,000 ng [a*]	ENA	101
		>20,000 ng [a*]	ENA1	
		>20,000 ng [a*]	ENA2	
		20 ng [a]	ENB1	
	Bifidobacterium bifidum 870T	>20,000 ng [a*]	ENA	
		20,000 ng [a]	ENA1	
		>20,000 ng [a*]	ENA2	
		20,000 ng [a]	ENB1	
	Bifidobacterium breve 4839T	>20,000 ng [a*]	ENA	
		20,000 ng [a]	ENA1	
		>20,000 ng [a*]	ENA2	
		>20,000 ng [a*]	ENB1	
	Bifidobacterium longum 4551	>20,000 ng [a*]	ENA	
		20,000 ng [a]	ENA1	

		>20,000 ng [a*]	ENA2	
		20,000 ng [a]	ENB1	
	Lactobacillus casei 4180	>20,000 ng [a*]	ENA	
		20,000 ng [a]	ENA1	
		>20,000 ng [a*]	ENA2	
		>20,000 ng [a*]	ENB1	
	Lactobacillus casei- casei 4180	>20,000 ng [a*]	ENA	
		20,000 ng [a]	ENA1	
		>20,000 ng [a*]	ENA2	
		20,000 ng [a]	ENB1	
	Lactobacillus rhamnosus 278T	>20,000 ng [a*]	ENA	
		20,000 ng [a]	ENA1	
		>20,000 ng [a*]	ENA2	
		>20,000 ng [a*]	ENB1	
	Lactobacillus ruminis 4061 T	>20,000 ng [a*]	ENA	
		20,000 ng [a]	ENA1	
		>20,000 ng [a*]	ENA2	
		20,000 ng [a]	ENB1	
	Streptococcus thermophilus 986	>20,000 ng [a*]	ENA	
		20,000 ng [a]	ENA1	
		>20,000 ng [a*]	ENA2	
		2,000 ng [a]	ENB1	
Protozoa	*Leishmania donovani* ATCC 39930D	10 µg/ml [b]	ENNs mix (A, A1, B, B1, B2 and Q)	118
	Plasmodium falciparum K1	0.27 µg/ml [b]	ENB	120
		0.20 µg/ml [b]	ENB4	
		1.1 µg/ml [b]	ENC	
		0.46 µg/ml [b]	ENG	
		1.9 µg/ml [b]	ENH	
		0.24 µg/ml [b]	ENI	
Fungi	Yeast — *Candida albicans* ATCC 90028	3.13 µg/ml [a]	ENA	195
		6.25 µg/ml [a]	ENA1	

			6.25 μg/ml [a]	ENB1	
		Saccharomyces cerevisiae 7	2000 ng [a]	ENA	101
			>20,000 ng [a*]	ENA1	
			>20,000 ng [a*]	ENA2	
			>20,000 ng [a*]	ENB1	
		Saccharomyces cerevisiae 15	>20,000 ng [a*]	ENA	
			>20,000 ng [a*]	ENA1	
			2000 ng [a]	ENA2	
			>20,000 ng [a*]	ENB1	
	Aspergillus flavus CECT 2684		>20 μg [a*]	ENB	100
	Aspergillus flavus CECT 2685		>20 μg [a*]		
	Aspergillus fumigatus CECT 20366		>20 μg [a*]		
	Aspergillus ochraceus CECT 2092		>20 μg [a*]		
	Aspergillus parasiticus CECT 2680		>20 μg [a*]		
	Beauveria bassiana CECT 20499		5 μg [a]		
	Beauveria bassiana CECT 20191		5 μg [a]		
	Beauveria bassiana CECT 20412		5 μg [a]		
	Fusarium oxysporum CECT 2715		>20 μg [a*]		
	Fusarium poae CECT 20165		>20 μg [a*]		
	Fusarium proliferatum CECT 20569		>20 μg [a*]		
	Fusarium proliferatum CECT 50546		>20 μg [a*]		
	Fusarium sporotrichioides CECT 20166		>20 μg [a*]		
	Fusarium tricinctum CECT 20150		>20 μg [a*]		
	Fusarium verticilloides CECT 2152		>20 μg [a*]		
	Fusarium verticilloides CECT 2982		>20 μg [a*]		
	Fusarium verticilloides CECT 2987		>20 μg [a*]		
	Penicillium expansum CECT 2275		>20 μg [a*]		
	Penicillium expansum CECT 2278		>20 μg [a*]		
	Trichoderma harzianum T22		1 μg [a]		
	Fusarium avenaceum		100 mg/kg [a]	ENB	65
	Fusarium graminearum		50 mg/kg [a]		
	Aspergillus fumigatus ATCC 90906		>50 μg/ml [a*]	ENA	195
			>50 μg/ml [a*]	ENA1	

		>50 µg/ml [a]*	ENB1	
	Botrytis cinerea	>100 µg/ml [a]	ENB	196
		75 µg/ml [a]	ENB1	
		>100 µg/ml [a]	ENB2	
		>100 µg/ml [a]	ENB4	
		>100 µg/ml [a]	ENJ1	
		>100 µg/ml [a]	ENJ4	

Supplementary Table S2: Effects of ENNs on insects.

* = not sensitivity at the highest test concentration; a = MIC value.

**Species**	**Sensitivity**	**ENN type**	**Reference**
Aphis craccivora	10 – 500 mg/L of *Cordyceps fumosorosea* micelial extract	ENK1	149
Bemisia tabaci	10 – 500 mg/L of *Cordyceps fumosorosea* micelial extract	ENK1	
Tenebrio molitor	> 14.26 µg/g[a]	Enniatin complex	150
Galleria mellonella	100 µg/g*	ENB	148
Choristoneura fumiferana	400 µg/g[a]	ENA/A_1	147
Aedes aegypti	10-75 µg/ml[a]	ENA / Enniatin complex	146
Calliphora erythrocephala	5-10 µg/fly[a]	ENA / Enniatin complex	
